# Sensing of chemical oxygen demand (COD) by amperometric detection—dependence of current signal on concentration and type of organic species

**DOI:** 10.1007/s10661-023-11228-3

**Published:** 2023-05-02

**Authors:** Samira Lambertz, Marcus Franke, Michael Stelter, Patrick Braeutigam

**Affiliations:** 1 Institute for Technical Chemistry and Environmental Chemistry, Friedrich Schiller University Jena, Philosophenweg 7a, 07743 Jena, Germany; 2 Center for Energy and Environmental Chemistry, CEEC Jena), Friedrich Schiller University Jena, Philosophenweg 7a, 07743 Jena, Germany; 3 Fraunhofer IKTS, Fraunhofer Institute for Ceramic Technologies and Systems, Michael-Faraday-Straße 1, 07629 Hermsdorf, Germany

**Keywords:** Chemical oxygen demand (COD), Sensor, Amperometric detection, Oxidation of organics, Wastewater treatment

## Abstract

The standard method to determine chemical oxygen demand (COD) with K_2_Cr_2_O_6_ uses harmful chemicals, has a long analysis time, and cannot be used for on-site online monitoring. It is therefore necessary to find a fast, cheap, and harmless alternative. The amperometric determination of COD on boron-doped diamond (BDD) electrodes is a promising approach. However, to be a suitable alternative, the electrochemical method must at least be able to determine the COD of water samples independently of the contained substances. Therefore, the current signal as a function of various organic materials was investigated for the first time. It was shown that the height of the signal current depended on the type of organic matter in single-substance solutions and that this substance dependency increases with the amount of COD. Those findings could be explained by the mechanism proposed for this reaction, showing that the selectivity of the reaction depends on the ratio of the concentration of hydroxyl radicals and organic species. We give an outlook on how to improve the method in order to increase the linear working range and avoid signal variance and how to further explain the signal variance.

**Supplementary Information:**

The online version contains supplementary material available at 10.1007/s10661-023-11228-3.

## Introduction

The ongoing industrialization, increased use of chemical products, and climate crisis are all factors potentially leading to water-related conflicts in the long run and pose a threat to natural water bodies (Goel, 2006). One way to face this potential danger and protect water as a natural resource is water monitoring and treatment (Quevauviller et al., 2007; Shannon et al., 2008). However, many classical methods of wastewater analysis use toxic chemicals, which leads to an increase in waste generation, analysis costs, and specialized personnel required for performing the analytical procedure.

An important sum parameter that is widely used in water monitoring is COD (Pisarevsky et al., 2005). COD measures the amount of oxygen that is needed to mineralize the organic content of a given water sample defined by standard reaction conditions. It can be used to monitor the quality of water bodies, access the degree of contamination in wastewater, and control wastewater treatment plants (Awe et al., 2016).

The standard method to determine COD uses potassium dichromate (K_2_Cr_2_O_6_) or potassium permanganate (KMnO_4_) as an oxidant (Boyles, 1997). Both are toxic and harmful to the person using it and the environment. The usage of K_2_Cr_2_O_6_ is likely to disappear in the future, since it is damaging to the environment and therefore the subject of several regulations (ECHA, 2015). In addition, the standard method uses other toxic chemicals (HgSO_4_, H_2_SO_4_, Ag_2_SO_4_), consumes high amounts of energy due to a long heating time, and is time-consuming (~4 h), making it unsuitable for in-time measurements and also resulting in a higher negative impact on climate change (Li et al., 2018).

COD is mentioned as a mandatory parameter in various laws and is therefore part of standard procedures in many countries. The German federal law states that COD must be measured whenever wastewater is discharged into surface waters (Ordinance on Requirements for the Discharge of Wastewater into Waters, German Federal Law, 1997). Similar laws can be found in various other states (e.g., USA, EU) (Urban Waste Water Treatment Directive, Annex I: Discharge requirements, 1991; US EPA The Water Quality Standards Regulation, 1983). Therefore, it is necessary to find a sustainable alternative to the standard method that can also be used in other applications like real-time monitoring and control of sewage treatment plants (Geerdink et al., 2017).

One way to avoid the use of toxic chemicals is to switch to methods that work without toxic chemicals, such as optical or electrochemical methods (Su et al., 2007). Among the research that has been conducted in finding alternative methods for the determination of COD, there have been non-oxidative and oxidative methods. A non-oxidative method is the spectrophotometric method which correlates the absorbance of water samples with COD. However, in this case, COD is only determined indirectly, the method has to be calibrated and no conclusion about the actual oxidizability can be drawn. The spectrophotometric method depends on the correlation between the adsorbance at a certain wavelength and COD (Mrkva, 1983). While a good correlation between UV absorbance and COD is shown for certain substances (lignin and humic substances, phenolic wastes), no conclusion on the actual oxidizability of the water samples can be made.

The wide range of oxidation-based methods that have been investigated includes some that are based on advanced oxidation processes (AOPs): the photocatalytic method, the photoelectrocatalytic method, and the electrocatalytic method (Li et al., 2018).

For the electrocatalytic method, research is mainly focused on finding suitable electrode materials (Gutierrez-Capitan et al., 2015; Cheng et al., 2011; Zhou et al., 2012) and establishing an electrochemical method. The most promising electrode material for the determination of COD is BDD, which has been investigated by several groups (Bogdanowicz et al., 2012) (Kondo et al., 2016; Wang et al., 2012a, b; Yu et al., 2007, 2009; Kondo et al., 2014). BDD has the advantage of a higher overpotential for oxygen evolution than other conventional electrode materials. (Panizza & Cerisola, 2009) Its potential for oxygen evolution reaction is 2.3 V vs SHE as compared to 1.9 V vs SHE for PbO_2_, which has the second highest potential. The electrochemical determination of COD on BDD electrodes is based on the oxidation of organic compounds by hydroxyl radicals produced on the electrode surface which have a high overpotential for oxygen evolution (Chang et al., 2008). Those hydroxyl radicals are supposed to react with organic matter in an unselective way, making it possible to determine all kinds of organic substances (Lee & Von Gunten, 2010).

Methods that have been used for the determination of COD with BDD electrodes are amperometric and coulometric methods. The amperometric method is more applicable for real-time analysis as it has a much shorter analysis time. It is instrumentally simple and fast and does not use any toxic chemicals. It can easily be automated and thus avoids high staff costs while making it usable for wastewater treatment plant monitoring and control. It has already been explored in several studies (Wang et al., 2012a, b; Yu et al., 2007, 2009). A summary of the studies investigating this approach with their stated working range, the chemicals used to validate the method, and some comments on why this is not applicable to real-world analysis can be found in Table 1.

**Table 1:** Published amperometric methods using BDD electrodes with their corresponding linear working range, studied substances, and comments

Authors	Linear working range	Substances studied	Comments
Yu et al. (2007)	20–9000 mg/L COD	Glucose, KHP, glutamic acid, phenol, p-nitrophenol, salicylic acid, cysteamine, oxalic acid, acetic acid	• Calibration was only conducted between 0 and 120 mg/L • Validation with the same substances that were used for calibration
Yu et al. (2009)	2–175 mg/L COD	Glucose, KHP, glutamic acid, phenol, p-nitrophenol, salicylic acid, cysteamine, oxalic acid, acetic acid	• Validation with the same substances that were used for calibration • Validation only in the range of 0–100 mg/L
Wang et al. (2012a)	19.2–11,600 mg/L COD	Glucose, KHP	• Calibration was only conducted between 0 and 120 mg/L • Only two organic substances were used
Wang et al. (2012b)	0–23,200 mg/L COD	Glucose, KHP	• Calibration was only conducted between 0 and 250 mg/L • Only two organic substances were used

While these papers show satisfactory results, one can criticize that it was not considered if there is a dependency of the signal current on the organic species. Only a few chemicals were used to evaluate the method. The methods were optimized and calibrated using one organic substance or a mixture of different organic substances with a constant composition. No attention was paid to the dependence of the signal on the composition of the sample. This means that the established methods are only applicable to the tested substances and samples. As COD is a sum parameter, it should be applicable to all water contents that are oxidizable by strong oxidants like K_2_Cr_2_O_6_. Until now, it is unknown if it is possible to analyze COD independently from the organic substance.

Therefore, the novelty of this study is the detailed investigation of the dependency of the electrochemical determination of BDD on the organic substance which has not been done for any electrode material and is mandatory for the understanding of the applicability of the method.

The aim of this study is to advance the research on the amperometric determination of COD with BDD electrodes to make it suitable for water monitoring systems and for the monitoring and management of wastewater treatment plants.

A necessary step towards establishing the proposed method in real-life applications is to investigate the dependency of the organic substance on the signal current as it should be applicable to all kinds of wastewaters with different compositions. Therefore, the dependence of the signal current on the organic species was investigated for the first time. Six diverse organic compounds (citric acid, glucose, acidic acid, sucrose, ascorbic acid, malonic acid) were investigated over a wide COD range (10 mg/L to 10,000 mg/L). They were used in single-substance samples to avoid leveling out of the signal variation between different compounds. Statistical methods (analysis of variance (ANOVA), Tukey’s test) and non-linear regression were used to further describe the concentration-dependent signal variation. A model was created explaining the signal variation depending on the concentration using the underlying reaction mechanism, which showed that the signal variation depends on the ratio of the concentration of hydroxyl radicals and organic substances. Based on these results, possible improvements in the method were derived. Finally, a calibration of the method was conducted to find out in which working range and with which precision the method can be used.

## Material and methods

### Chemicals and samples

All chemicals were used as provided and are listed in Table 2 including provider and purity.

**Table 2:** Used chemicals and specifications

Chemical	Purchased by	Purity
Na_2_SO_4_	Merck	≥ 99%
H_2_SO_4_	VWR	100%
D(+)-Glucose monohydrate	Carl Roth	≥ 99.5%
Citric acid	VWR	100%
Acidic acid	Merck	100%
D(+)-Sucrose	Roth	≥ 99.5%
L(+)-Ascorbic acid	Roth	≥ 99%
Malonic acid	Sigma-Aldrich	99%

All solutions were prepared using freshly filtered ultrapure water (σ ≤ 0.055 μS/cm, TOC < 5 ppb; GenPure Pro, Fisher Scientific). The electrolyte was prepared using 0.1 M Na_2_SO_4_ and 0.1 mM H_2_SO_4_.

For the preparation of COD samples, a 10,000 mg/L COD stock solution was prepared with the theoretical amount of organic species for the given COD. The calculation can be found in the Online Resources (S1). The organic species were dissolved in the electrolyte. For the mixture, a 10,000 mg/L COD stock solution was prepared containing 1666.6 mg/L COD from each species. All other COD sample solutions were prepared as dilutions from the stock solution with the electrolyte as a solvent. The solutions containing organic substances were stored at 7° C before usage.

### Experimental setup

**Fig. 1:**
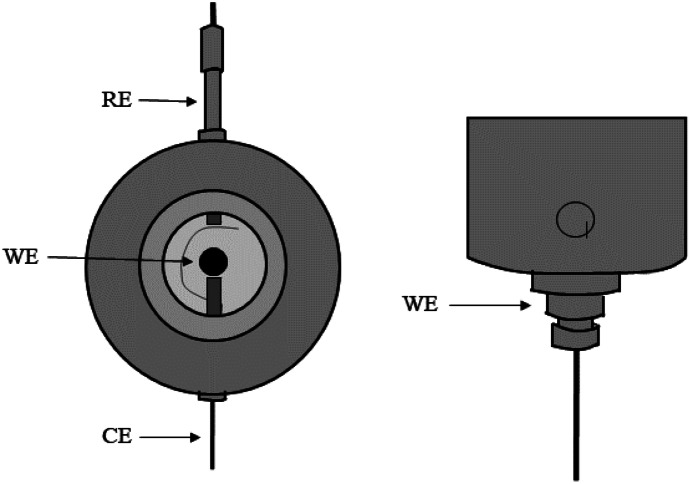
Three-electrode measurement cell containing working electrode (WE), reference electrode (RE), and counter electrode (CE)

The experimental setup consisted of a three-electrode measurement cell, a stirrer, and a potentiostat. The exact geometry of the custom-made Teflon electrochemical cell (Volume 17 ml) (Fig. 1) can be found in the Online Resources (S2).

A commercial BDD electrode (5 µm diamond on niobium, coated on both sides, 8 mm diameter, DiaCCon) was used as a working electrode, an Ag/AgCl (3 M NaCl) electrode (RE-1S reference electrode (Ag/AgCl), ALS) as a reference electrode, and a platinum wire as a counter electrode (diameter 0.5 mm, length 10 mm, purity 99.95%, Polymet). The geometry of the electrodes and their corresponding mounting can be found in Online Resources (S3). The electrochemical cell was connected to a potentiostat (Versastat 3F, Princeton Applied Research) for electrochemical measurements. The solution was stirred using a stirrer (IKA Nanostar 7.5 digital stirrer) that was connected to a custom agitator shaft. The exact geometry of the agitator shaft can be found in Online Resources (S4).

### Electrochemical method

All experiments were done following the same procedure. Before starting the measurement, the electrochemical cell was filled with 12 ml of electrolyte. The stirrer was started with a stirring speed of 100 rpm. The amperometric measurement method was started. It consisted of an activation step with a potential of 3 V vs Ag/AgCl (3 M NaCl) for 30 s. Subsequently, the potential was set to 2.4 V vs Ag/AgCl (3 M NaCl) for 170 s. After a waiting period of 110 s, 5 ml of the sample solution was added to the measurement cell. The current measured at 2.4 V vs Ag/AgCl (3 M NaCl) was used for the calculation of the signal current. All experiments were executed at room temperature in a triple determination.

### Data analysis

#### Calculation of the signal current

The current signal was calculated from the current–time curve using several data processing steps:

1. Smoothing of the data by using a moving average function and a window length of 20
2. Calculating the average over the background current
3. Subtracting the average of the background current from the signal current
4. Calculating the average of the reduced signal current

The smoothing of the data is used to average out signal noise from stirring, current anomalies, and gas evolution on the electrode.

The calculated average of the reduced signal current was used as a current signal. The data processing was done using a KNIME® workflow that can be found in the Online Resources (S5).

### Statistical methods

The statistical calculations that were used in the data evaluation were done with OriginLab Pro. An ANOVA was used to evaluate if the signal current for different organic substances showed a significant difference. Subsequently, the Tukey test was used for those data sets that showed a significant difference between the groups. It determined which of the groups exactly showed the significant difference, resulting in subgroups with similar mean values. All tests were done using a significance level of 0.05. The calibration curve was evaluated comparing the adjusted *R*^2^ for different concentration ranges. The concentration range with the highest adjusted *R*^2^ was chosen as the working range.

## Results and discussion

### Dependence of the electrochemical oxidation at BDD electrodes on the type of organic compound

A necessary step in establishing the amperometric method for determining COD is to check whether there is a dependence of the current signal on the organic substances in the water sample. Since COD is a sum parameter, it must be possible to apply the method independently from all water constituents and thus the current signal must be independent of the organic substances. The compounds used here were selected from a wide range of chemicals used in COD sensor studies and derived substances. The final substances were selected from a larger group of chemicals by preliminary testing to achieve the greatest possible variation between signals. Measurements were made using sample solutions of each compound. In a real water sample, a mixture of different organic compounds and other species is present. This could lead to a compensation of the signal between different species which is avoided here by using single compound samples to simulate extreme situations where only readily or poorly oxidizable compounds are present. No non-organic water components were used in order to avoid overlapping effects on the signal current, since this study focuses only on the organics.

**Fig. 2:**
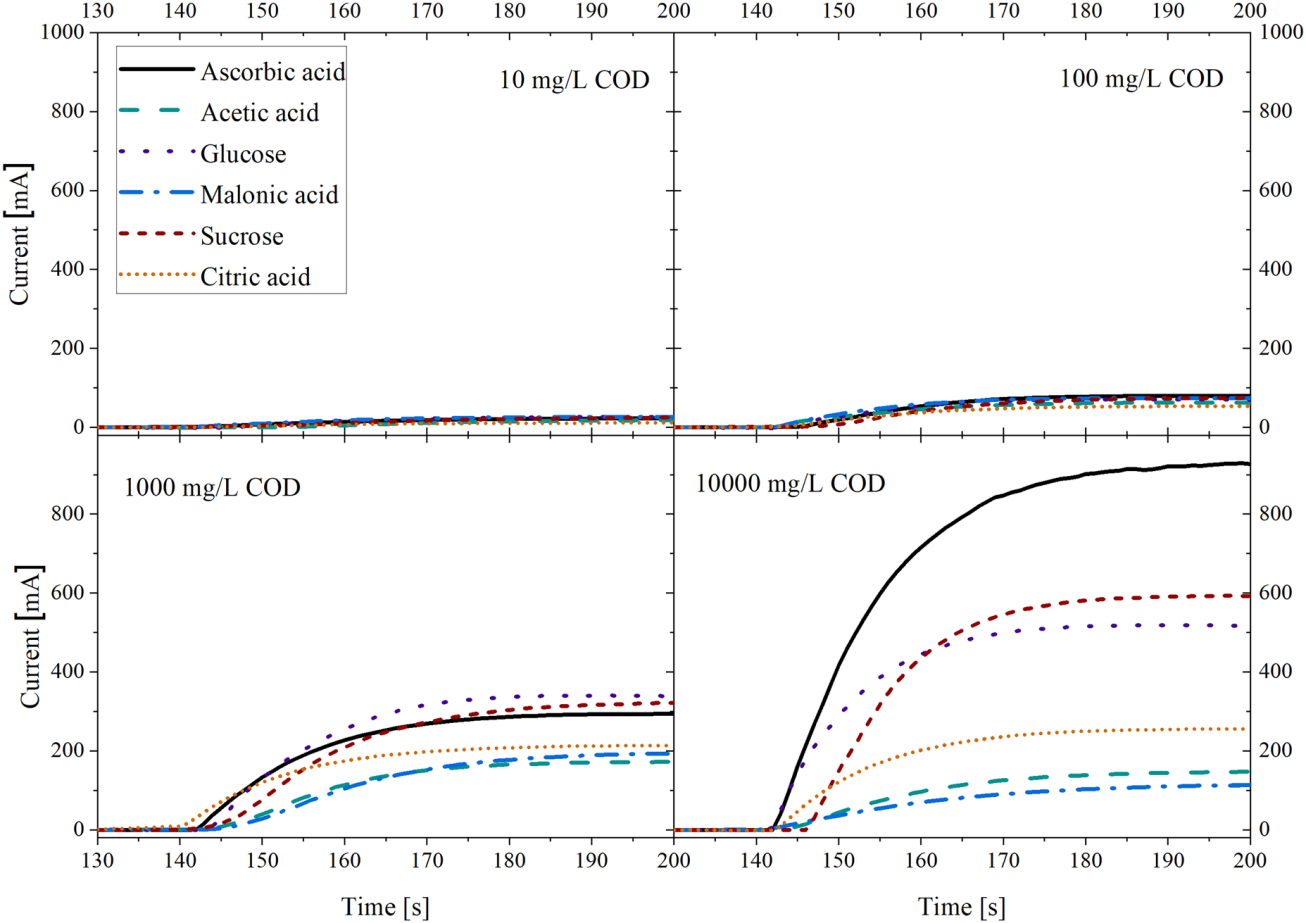
Current–time curves for the described electrochemical method at different CODs (10 mg/L, 100 mg/L, 1000 mg/L, 10,000 mg/L) and for different organic compounds (ascorbic acid, acetic acid, glucose, malonic acid, sucrose, citric acid)

To determine the dependence of the electrochemical oxidation at BDD electrodes on the type of organic compounds, current–time curves were recorded for six distinct species (ascorbic acid, acetic acid, glucose, malonic acid, sucrose, citric acid) at four different COD values (10 mg/L, 100 mg/L, 1000 mg/L, 10,000 mg/L) (Fig. 2).

Figure 2 shows that not only the absolute value of the current signal increases as one would expect for a concentration-dependent sensing method but also does the variation between current values for different organic species at different COD values. While the variation between current signals is low at 10 mg/L and 100 mg/L, there is a visible deviation at 1000 mg/L and even more at 10,000 mg/L.

**Fig. 3:**
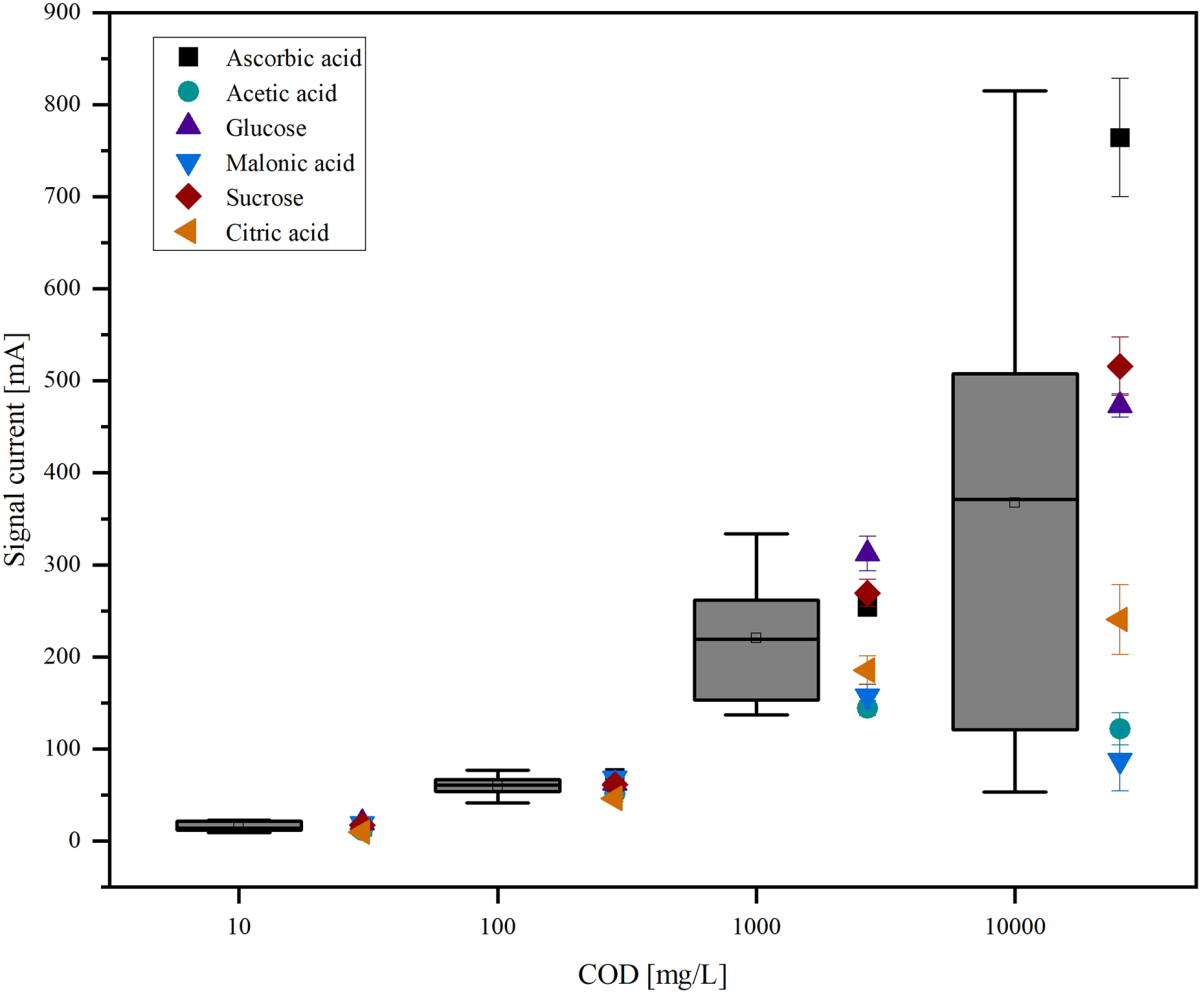
Signal currents for several organic species (ascorbic acid, acetic acid, glucose, malonic acid, sucrose, citric acid) at different COD values (10 mg/L, 100 mg/L, 1000 mg/L, 10,000 mg/L). The signal currents are shown as a boxplot and as individual measurements

The calculated current signal for the different organic species at different COD values is shown in Fig. 3.

The average current signal increases with increasing COD but so does the range of current signals and the deviation between the signal for different species.

A one-way ANOVA was performed to evaluate if the difference between the current signal of distinct species is significant at different COD values. The detailed ANOVA calculations can be found in Online Resources (S6).

The ANOVA calculations show that the difference between the average signal current is not significant for 10 mg/L COD (*p* = 0.05). For all the other COD values, there is a significant difference between current signals.

Subsequently, a Tukey test was conducted for current signals at 100 mg/L, 1000 mg/L, and 10,000 mg/L. While ANOVA only determines if there is a significant difference between the compared groups (in this case the different organic compounds), with the Tukey test groups that show a significant difference can be found at the stated significance level (*p* = 0.05).

The resulting groups of organic compounds with no significant difference in the current value are listed in Table 3.

**Table 3:**
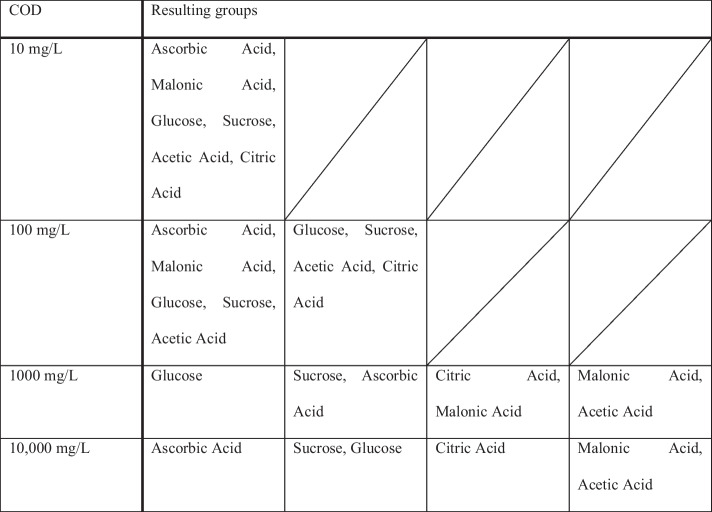
Groups with significantly different signal currents resulting from the Tukey test with a significance level of 0.05

For the 100 mg/L COD samples, two groups can be built that either contain all the compounds but citric acid or all the compounds but malonic acid and ascorbic acid. For 1000 mg/L COD and 10,000 mg/L COD, the comparison of groups showed that higher oxidized compounds that only contain carboxylic groups (citric acid, malonic acid, acetic acid) were clustered together as were the lower oxidized compounds that also contain other functional groups (ascorbic acid, glucose, sucrose).

The data shows that the current signal is highly dependent on the organic species and the difference between the current signals increases with increasing COD, meaning that the dependency can be described by different functions for different organic substances. The variation between different organics is significant for all COD values above 100 mg/L. The Tukey test was used to divide data into groups containing the compounds with significantly different current signals. The groups can be used to draw a preliminary conclusion on whether the chemical structure of the organic compound influences the current signal, although this is very limited due to the small sample size. For 100 mg/L, only citric acid shows a lower current signal, which could be explained by the fact that it is already highly oxidized. In contrast, malonic acid and ascorbic acid show a higher signal current compared to the other compounds. They have quite different structures, so no statement can be made at this point as to why they differ so much. For 1000 mg/L and 10,000 mg/L, it is not possible to find a correlation between the structure and the average current, since the compounds used are quite different. It is noteworthy that the average current for acetic acid, malonic acid, and citric acid does not increase significantly from 1000 to 10,000 mg/L COD. This could mean that the electrode is already saturated at 1000 mg/L COD.

### Concentration-dependent current signal from the electrochemical oxidation of different organic species at BDD electrodes

The course of the current signal over a wide COD range should be related to the mechanism of the oxidation of organic compounds at BDD electrodes.

It is known that the oxidation of organic compounds at BDD electrodes follows a two-step mechanism (Martinez-Huitle & Ferro, 2006):

1 $$\mathrm{BDD}()+{\mathrm{H}}_{2}\mathrm{O}\leftrightharpoons \mathrm{BDD}\left(\mathrm{OH}\right)+ {\mathrm{H}}^{+}+ {\mathrm{e}}^{-}$$

2 $$\mathrm{BDD}\left(\mathrm{OH}\right)+\mathrm{R }\to \mathrm{BDD}()+{\mathrm{CO}}_{2}+ {\mathrm{H}}_{2}\mathrm{O}$$

In the first step, hydroxyl radicals are formed from water at the BDD electrode (Eq. 2), which then react with an organic compound in the second step (Eq. 3). When the organic compound is completely oxidized, the reaction products are CO_2_ and water. From this mechanism, an equation describing the concentration dependence of the current can be derived under various assumptions (Popović & Johnson, 1998):

3 $${\mathrm I}_{\mathrm{tot}}\;=\;2\mathrm z\mathrm F\mathrm\Gamma{(\mathrm k}_2{\mathrm c}_{\mathrm R}\cdot{\mathrm k}_1)/({\mathrm k}_2{\mathrm c}_{\mathrm R}+{\mathrm k}_1)$$

Here, *k*_1_ is the reaction constant for the forward reaction of Eq. 2, while *k*_2_ is the reaction constant of Eq. 3. *c*_R_ is the concentration of the organic species, *z* is the number of electrons transferred, *Γ* is the coverage of the electrode, and *F* is the Faraday constant. *z* and *Γ* are assumed to be constant during the process.

**Fig. 4:**
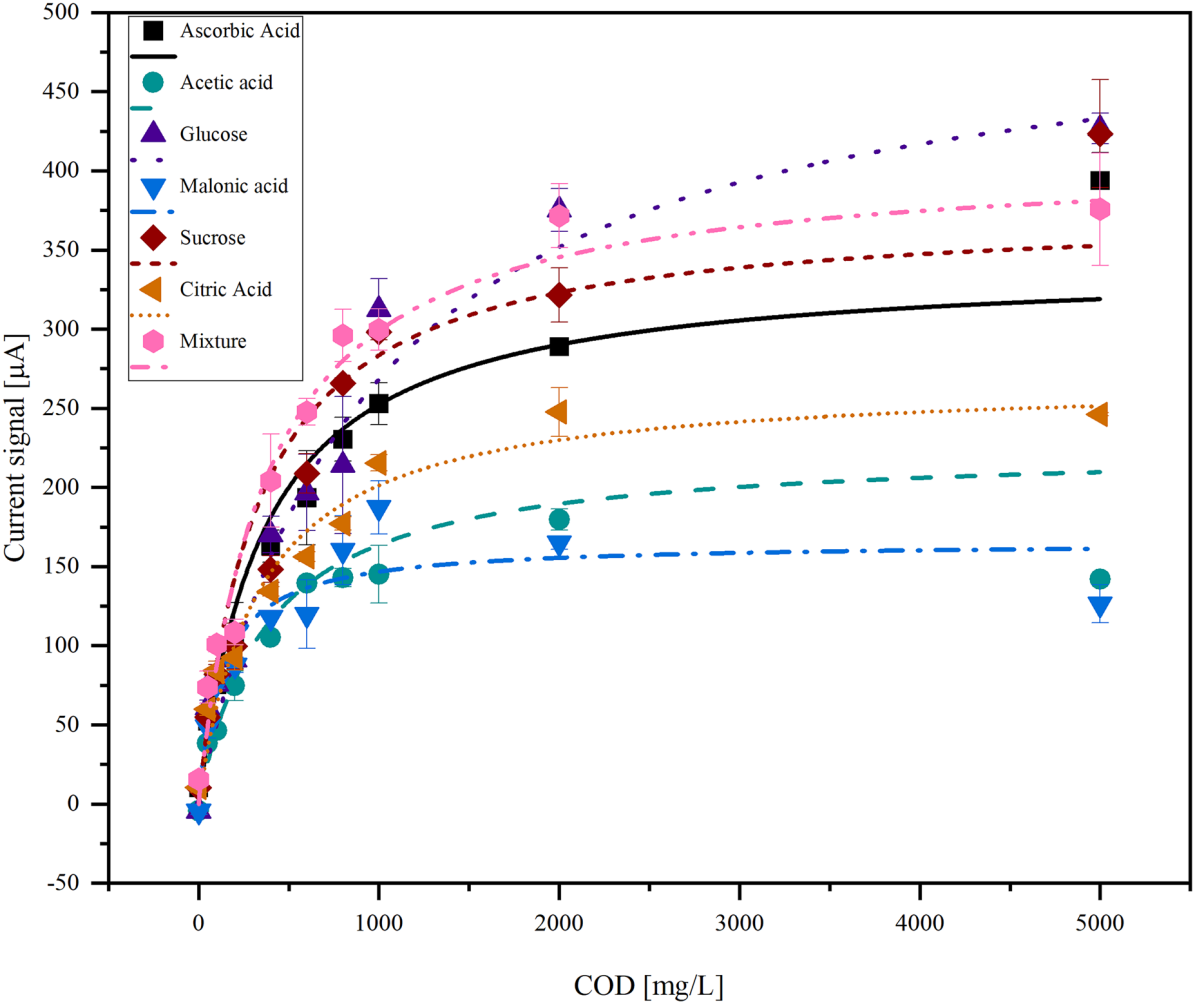
COD dependency of the current signal for different organic species (acetic acid, glucose, malonic acid, ascorbic acid, sucrose, citric acid) and a mixture of the compounds in the COD range from 0 mg/L to 5000 mg/L

To investigate the course of the current signal over a wide COD range, it was recorded depending on the COD for different organic compounds (Fig. 4).

Figure 4 shows that the course of the curve is dependent on the organic species, and that it is not linear over the whole COD range investigated here.

Equation 3 was simplified to Eq. 4, which was used to fit the data in Fig. 4.

4 $$y=a{(k}_2\mathrm x\cdot k_1)/(k_2\mathrm x+k_1)$$

*a*, *k*_1_, and *k*_2_ were calculated from the fit data (Table 4).

**Table 4:** Fit coefficients from the non-linear fit of the COD-dependent signal current

Organic compound	*a*	*k*_1_	*k*_2_
Acetic acid	0.0025	0.089	2.36 × 10^−4^
Glucose	0.0058	0.088	9.74 × 10^−5^
Malonic acid	0.0062	0.027	2.49 × 10^−5^
Ascorbic acid	0.0027	0.125	3.55 × 10^−4^
Sucrose	0.0058	0.064	1.97 × 10^−4^
Citric acid	0.0055	0.048	1.46 × 10^−4^
Mixture	0.0027	0.152	4.12 × 10^−4^

While *a* and *k*_1_ are in the same order of magnitude for all organic compounds, *k*_2_ varies over different orders of magnitudes.

**Fig. 5:**
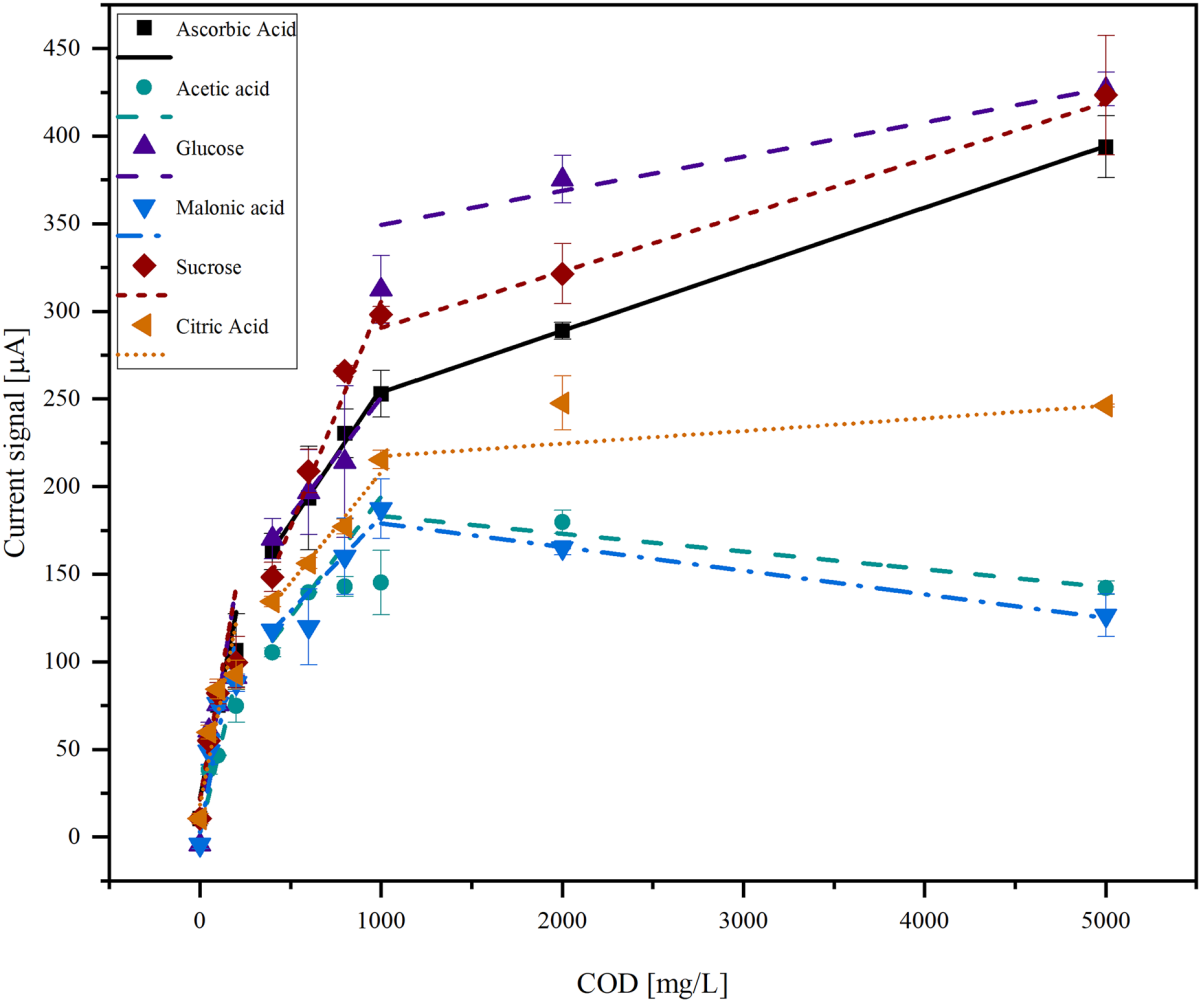
COD dependency of the current signal for different organic species (acetic acid, glucose, malonic acid, ascorbic acid, sucrose, citric acid) in the COD range from 0 to 5000 mg/L. The data was fitted linearly

The data from Fig. 4 can also be fitted linearly. Therefore, it is divided into three parts (Fig. 5).

In the first area between 0 and 100 mg/L COD, the linear curves for all organic compounds overlap. In the second area between 100 and 1000 mg/L COD, the curves drift apart and show a significant dependence on the organic species. In the area between 1000 and 10,000 mg/L, the variance between different organic species becomes even bigger.

The large degree of congruence of the data with the fit is an indication that the reaction taking place can be described well by the mechanism. However, the fit only works for data up to 2000 mg/L. This could be due to the assumptions made in deriving the equation, which do not work for higher concentrations. One of the assumptions is the steady state for the formation of the hydroxyl radical. At high concentrations of organic compounds, the reaction of hydroxyl radicals with organic compounds is so fast that they cannot be replaced immediately by new ones. This could be the reason why the equation does not work at higher concentrations.

The fitting parameters *a*, *k*_1_, and *k*_2_ describe the prefactor and kinetic constants of the reaction, respectively. *a* and *k*_1_ should be similar for all organic compounds because they describe the formation of hydroxyl radicals on the BDD surface, which is independent of the organic compound. As expected, they are of the same order of magnitude for all organic compounds. *k*_2_ describes the reaction between the hydroxyl radical and the organic compound. This reaction is selective and *k*_2_ varies between different orders of magnitude. The linear fit of the data shows different linear ranges. Each of them is dominated by a different reaction:

Small concentrations: $${k}_{1}\gg {k}_{2}{c}_{\mathrm{R}}$$

The determination of COD is independent of the type of organic species. The excess of produced hydroxyl radicals at the electrode over the organic species is so high that every molecule reaching the electrode is directly oxidized. The reaction rate depends only on the total concentration of organic species since this determines the rate of hydroxyl radical production. It is possible to determine the COD independently from the species contained in this range.

Medium concentrations: $${k}_{1}\approx {k}_{2}{c}_{\mathrm{R}}$$

The total reaction constant is affected by both processes: the production of the hydroxyl radical and the reaction of the organic species with the hydroxyl radical. Since the rate of hydroxyl radical production is similar to the rate of reaction with the organic species and there is no excess of either process, the reaction is selective towards different organic compounds and the different species show different current signals. It is not possible to determine the COD independently from the substances contained in this range, but it can be determined if a calibration for the specific composition is available.

High concentrations: $${k}_{1}\ll {k}_{2}{c}_{\mathrm{R}}$$

The concentration of organic species is so high that any hydroxyl radical generated is immediately consumed, and the generation of hydroxyl radicals becomes the rate-determining step. The current is independent of the concentration of organic species and does not increase with the amount of organic species present because the electrode is already saturated. It is not possible to determine the COD in this range.

**Fig. 6:**
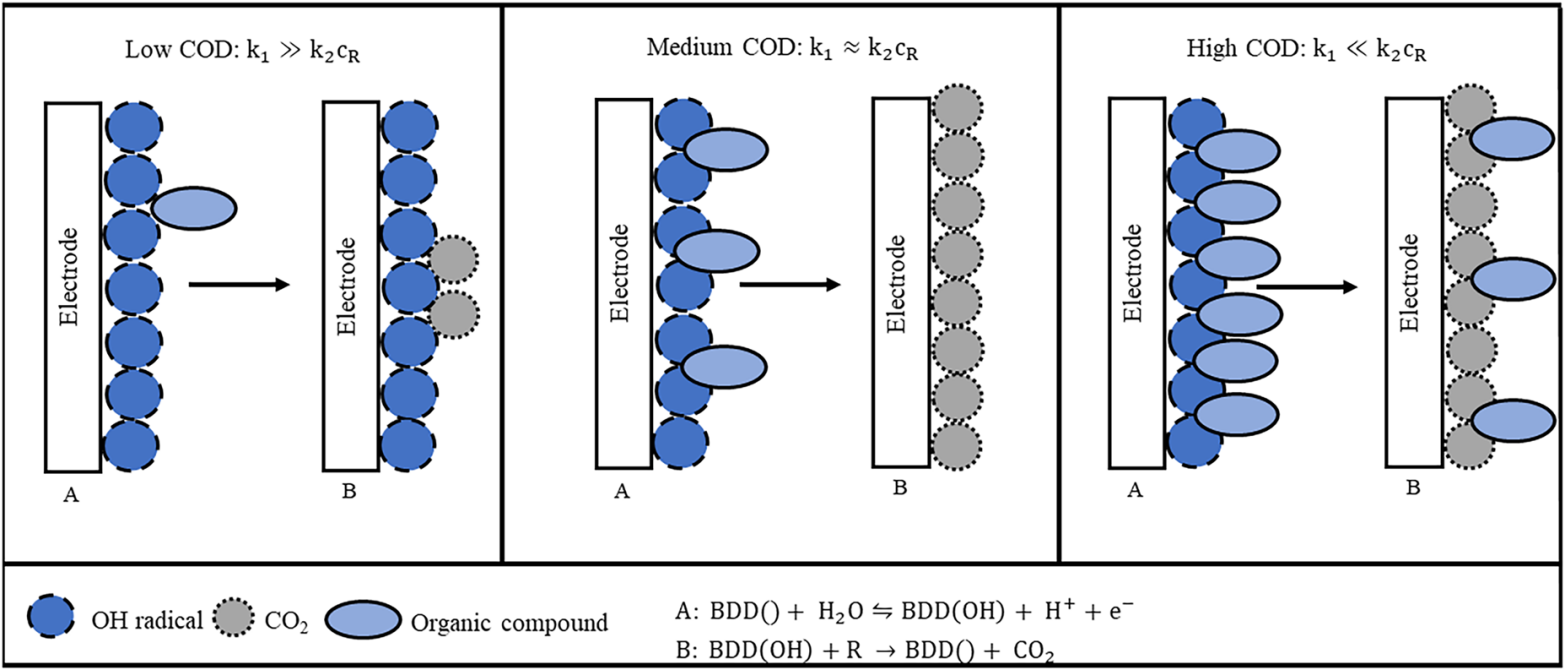
Processes at the electrode dependent on the amount of organic species in the wastewater sample for a low COD, medium COD, and high COD. The electrode is shown after the formation of hydroxyl radicals and after the combustion of organic compounds

Figure 6 shows the processes taking place dependent on the concentration range.

While the mechanism used to describe the reaction is a good and simple approach for some general conclusions, several simplifications have been made. Important factors not considered in this simplification are the formation of radicals other than the hydroxyl radical and the incomplete oxidation of organic compounds.

Since different radicals have different oxidation potentials, lifetimes, and mobility within the cell, they can affect the selectivity of the reaction (Farhat et al., 2015; Lee & Von Gunten, 2010). A better knowledge of the type and amount of radicals formed could contribute to a better understanding of the reactions taking place and could be used to influence them. Thus, the dependence of the current signal on the organic species could be reduced.

The incomplete oxidation of organic compounds could also lead to an underestimation of COD. Theoretically, complete oxidation of the organic compounds to CO_2_ is expected in the mechanism, which is a prerequisite for the correct determination of COD. COD can only be determined correctly if all organic compounds and their intermediates are included in the reaction. If the final product for some compounds is not CO_2_, the COD will be underestimated (Mascia et al., 2007).

The data show that the oxidation of some compounds is easier than others. As shown in the literature, the relationship between the structure of a compound and its reaction can be modeled (Jiang et al., 2017). This would help to determine what types of wastewater can be examined using the method described. Knowing the relationship between structure and the signal current can also help optimize the method and could be used to predict its scope.

In summary, the results of this section show that the reaction occurring in the sensor can be described by the mechanism used in the literature, although there are limitations due to the simplifications used. The organic compounds are oxidized by the hydroxyl radicals electrochemically formed on the BDD surface. The change in signal current depends on the ratio between organic compounds and hydroxyl radicals. The method only works independently of the organic species if the concentration of the organic compounds is much lower than the amount of hydroxyl radicals formed.

### Determination of the working range for the determination of COD independently of the organic species

**Fig. 7:**
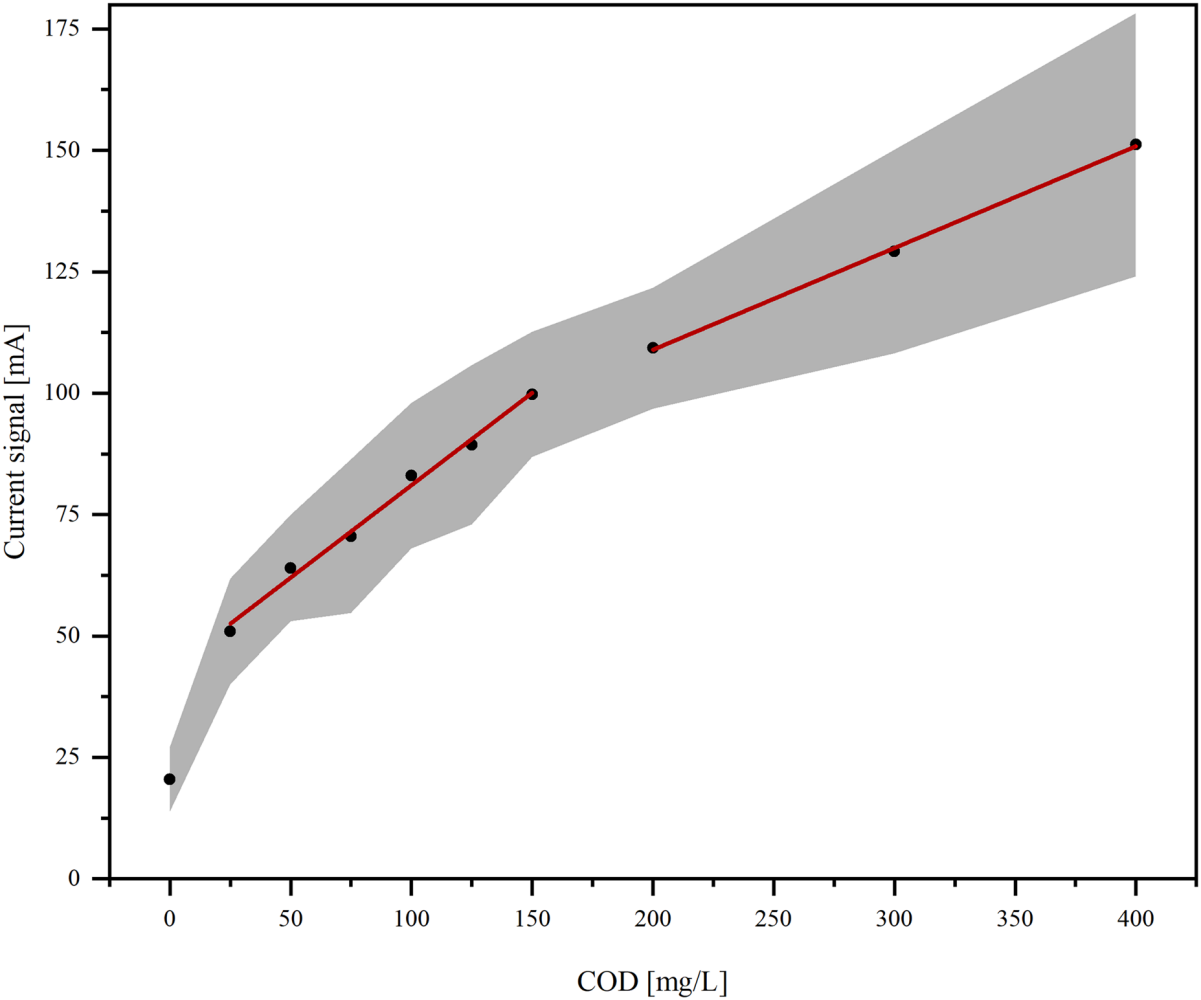
Calibration curve for the determination of COD with the electrochemical method. The calibration curve was built using the average current of the individual measurements of all used organic species (black dots). The grey area shows the standard deviation of the individual measurements of all used organic species

To determine the working range in which the determination of COD is possible independently of the organic species, a calibration curve was created between 0 and 400 mg/L COD, using the mean and standard deviation of the signal current from the individual measurements of organic species (Fig. 7).

The calibration curve shows two linear working ranges for 25–150 mg/L and for 200–400 mg/L. The standard deviation for all organic compounds becomes larger at higher COD values because the dependence of the current signal on the type of organic species increases. Therefore, the higher linear working range for 200–400 mg/L is not included in the linear working range for compound-independent determination of the COD.

The precision of the calibration was calculated from the standard deviation of the measurements for all organic species. It was calculated by $p=s_{\mathrm y}/m\cdot100\%$ with the precision *p*, the standard derivation of the current signal *s*_y_, and the slope of the calibration curve *m*. This resulted in a precision of 30%. The precision of the method depends on the organic compounds in the water sample and cannot be used to describe the overall ability of the method.

Since the variation of the current signal for different organic species depends mainly on the ratio between the amount of hydroxyl radicals and organic compounds, one way to increase the linear range of the method is to increase the amount of hydroxyl radicals present while keeping the COD of the sample constant. In the context of general process optimization, this can be done by increasing the surface area of the electrode without increasing the volume of the measuring cell and the sample volume.

This can be done, for example, by using a porous working electrode, resulting in a larger active surface area on the same geometric area. Since the hydroxyl radicals are only on the electrode surface, more effective mixing would result in a higher ratio of hydroxyl radicals to organic compounds in the bulk medium. This could be achieved by using an ultrasonic mixer instead of a mechanical stirrer.

Similarly, it is possible to reduce the amount of organic compounds while maintaining the same amount of hydroxyl radicals. Therefore, a thin film cell with a minimal amount of sample would be a possible approach.

In summary, COD can be determined with a precision of 30% in the linear range of 25–150 mg/L, independent of the organic species for the used method with the used organic compounds.

## Conclusion

In this paper, the dependence of the current signal on the organic species in the amperometric determination of COD is described for the first time. The variation of the current signal depends on COD quantity and increases with increasing COD. While COD can be determined independently of the organic species up to about 150 mg/L, the dependence of the current signal on the organic species makes it impossible to determine COD above 150 mg/L independently of the organic species.

The dependence on organic species can be explained by the mechanism of the reaction, which consists of two reactions: the formation of hydroxyl radicals on the BDD surface and the reaction of hydroxyl radicals with the organic compounds. Depending on the amount of organic compounds in the measurement cell, one of the two reactions dominates. At low concentrations, there is an excess of hydroxyl radicals, so that any organic compound can be oxidized unselectively. At intermediate concentrations, the amount of hydroxyl radicals is similar to the amount of organic compounds, meaning that the reaction is selective. At high concentrations, there is an excess of organic compounds so that the electrode is saturated and the current does not increase with increasing concentration. To increase the working range in which all organic compounds can be oxidized non-selectively, the ratio between the electrode surface area and the COD must be increased.

To determine a compound-independent linear working range, the calibration curve was constructed from individual measurements of single-substance samples of various organic compounds. A linear working range of 25–150 mg/L can be achieved with a low detection limit of 25 mg/L and a precision of 30%.

## Supplementary Information

Below is the link to the electronic supplementary material.

Supplementary file1 (DOCX 490 KB)

## Data Availability

The data required to reproduce the above findings are available upon request.

## Abbreviations

COD: Chemical oxygen demand
BDD: Boron-doped diamond
ANOVA: Analysis of variance
AOP: Advanced oxidation process
