# Supplementary material for: Sensing of chemical oxygen demand (COD) by amperometric detection—dependence of current signal on concentration and type of organic species
Source: Environ Monit Assess. 2023 May 2;195(6):630. doi: 10.1007/s10661-023-11228-3 (PMC10154276; doi:10.1007/s10661-023-11228-3)
Supplement: Supplementary file 1 — Supplementary file1 (DOCX 490 KB) [file 10661_2023_11228_MOESM1_ESM.docx]

**Sensing of chemical oxygen demand (COD) by amperometric detection –**

**Dependence of current signal on concentration and type of organic species**

Environmental Monitoring and Assessment

Samira Lambertz, Marcus Franke, Michael Stelter, Patrick Braeutigam*

^*^ Corresponding author. Institute for Technical Chemistry and Environmental Chemistry, Center for Energy and Environmental Chemistry (CEEC Jena), Friedrich Schiller University Jena, Fraunhofer Institute for Ceramic Technologies and Systems. E-mail address: patrick.braeutigam@uni-jena.de.

## S1 Calculation of the necessary amount of organic species

1. Calculation of the oxygen equivalent $n$ for the complete combustion of the organic species:

$$C_{a}H_{b}O_{c}+n O_{2} \to x {CO}_{2}+y H_{2}O$$

$$n=\frac{2a+ \frac{b}{2}-\frac{c}{2}}{2}$$

1. Calculation of the necessary amount of organic species $m_{organic}$ [g] for a selected amount of COD and sample volume

$$m_{organic}=\frac{n \cdot COD}{32000 \cdot M_{organic}\cdot V}$$

with

$$COD:selecte amount of COD in\frac{mg}{L}$$

$$M_{organic}:molar weight for the selected organic species in g/mol$$

$$V:Sample volume in L$$

## S2 Exact geometry of the measurement cell


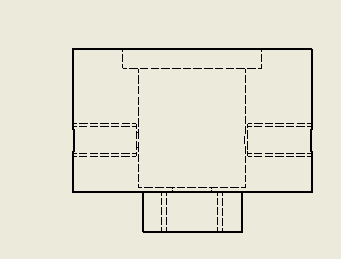


35 mm

5 mm

30 mm


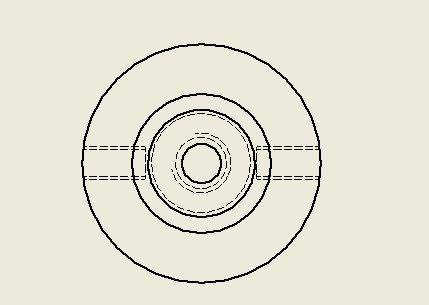


ø 10 mm

ø 7 mm

ø 7 mm

ø 27 mm

ø 35 mm

ø 3 mm

ø 60 mm

## S3 Exact geometry of the electrodes and their mounting

### S3.1 Working electrode

Seal ring


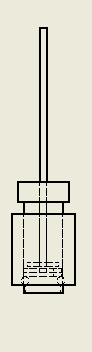


18 mm

26 mm

2 mm

10 mm

5 mm

BDD electrode and titanium plate

Silver wire

Flat seal


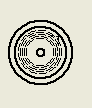


Ø 13 mm

Ø 15 mm

Ø 2 mm

### S3.2 Counter electrode


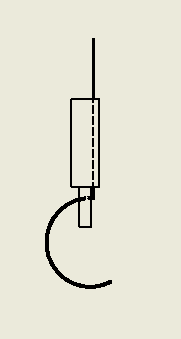


Platinum wire

22 mm

10 mm

3 mm


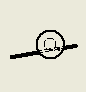


Ø 7 mm

### S3.3 Reference electrode


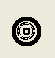


Ø 7 mm

Ø 4,2 mm


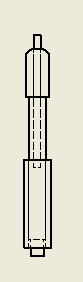


22 mm

Commercial reference electrode

## S4 Geometry of the stirrer


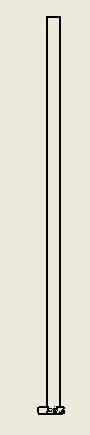


200 mm


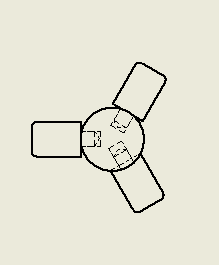


Ø 6,4 mm

3 mm

4,5 mm

## S5 Knime Workflow for Data analysis


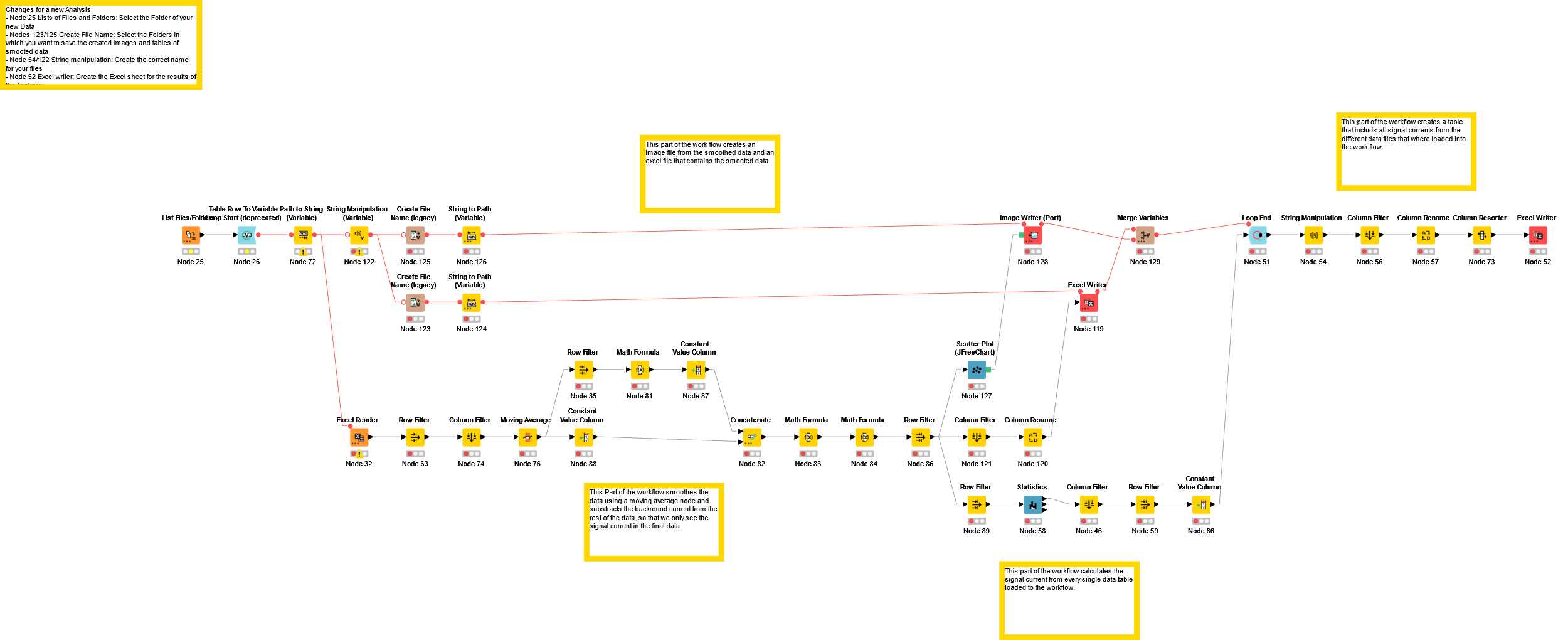


## S6 Data for the ANOVA at different COD values

### S6.1 COD = 10 mg/L

| Organic substance | Average value | Standard deviation | Standard error of the average value |
| --- | --- | --- | --- |
| Ascorbic acid | 1,51904E-5 | 2,76552E-6 | 1,59668E-6 |
| Acetic acid | 1,15531E-5 | 2,58459E-8 | 1,82758E-8 |
| Glucose | 1,96076E-5 | 2,53884E-6 | 1,79523E-6 |
| Malonic acid | 1,86538E-5 | 5,89055E-6 | 3,40091E-6 |
| Sucrose | 1,71102E-5 | 5,38545E-6 | 3,10929E-6 |
| Citric acid | 9,42514E-6 | 4,76569E-7 | 3,36985E-7 |

|  | DF | Sum of Squares | Average sum of squares | F-value | Probability > F |
| --- | --- | --- | --- | --- | --- |
| Model | 5 | 1,76455E-10 | 3,5291E-11 | 2,12635 | 0,15357 |
| Error | 9 | 1,49373E-10 | 1,6597E-11 |  |  |
| Total | 14 | 3,25828E-10 |  |  |  |

### S6.2 COD = 100 mg/L

| Organic substance | Average value | Standard deviation | Standard error of the average value |
| --- | --- | --- | --- |
| Ascorbic acid | 6,84634E-5 | 2,13436E-6 | 1,23227E-6 |
| Acetic acid | 5,14468E-5 | 8,83946E-6 | 5,10346E-6 |
| Glucose | 6,25174E-5 | 8,92918E-6 | 5,15526E-6 |
| Malonic acid | 6,79278E-5 | 7,93696E-6 | 4,58241E-6 |
| Sucrose | 6,09449E-5 | 4,18889E-6 | 2,41846E-6 |
| Citric acid | 4,62415E-5 | 1,29266E-6 | 7,46316E-7 |

|  | DF | Sum of Squares | Average sum of squares | F-value | Probability > F |
| --- | --- | --- | --- | --- | --- |
| Model | 5 | 1,20947E-9 | 2,41894E-10 | 5,93277 | 0,00548 |
| Error | 12 | 4,8927E-10 | 4,07725E-11 |  |  |
| Total | 17 | 1,69874E-9 |  |  |  |

### S6.3 COD = 1.000 mg/L

| Organic substance | | Average value | | Standard deviation | | Standard error of the average value | |
| --- | --- | --- | --- | --- | --- | --- | --- |
| Ascorbic acid | | 2,54134E-4 | | 9,73564E-6 | | 5,62088E-6 | |
| Acetic acid | | 1,44004E-4 | | 8,32407E-6 | | 4,8059E-6 | |
| Glucose | | 3,1242E-4 | | 1,88743E-5 | | 1,08971E-5 | |
| Malonic acid | | 1,57245E-4 | | 1,26806E-5 | | 7,32117E-6 | |
| Sucrose | | 2,69467E-4 | | 1,46553E-5 | | 8,46124E-6 | |
| Citric acid | | 1,85607E-4 | | 1,50867E-5 | | 8,7103E-6 | |
|  | DF | | Sum of Squares | Average sum of squares | F-value | | Probability > F |
| Model | 5 | | 6,91458E-8 | 1,38292E-8 | 73,85434 | | 1,36802E-8 |
| Error | 12 | | 2,24699E-9 | 1,87249E-10 |  | |  |
| Total | 17 | | 7,13928E-8 |  |  | |  |

### S6.4 COD = 10.000 mg/L

| Organic substance | Average value | Standard deviation | Standard error of the average value |
| --- | --- | --- | --- |
| Ascorbic acid | 7,64011E-4 | 6,42374E-5 | 3,70875E-5 |
| Acetic acid | 1,2157E-4 | 1,75441E-5 | 1,01291E-5 |
| Glucose | 4,72791E-4 | 1,27203E-5 | 7,34406E-6 |
| Malonic acid | 8,72927E-5 | 3,29194E-5 | 1,9006E-5 |
| Sucrose | 5,15462E-4 | 3,14928E-5 | 1,81824E-5 |
| Citric acid | 2,40594E-4 | 3,79886E-5 | 2,19328E-5 |

|  | DF | Sum of Squares | Average sum of squares | F-value | Probability > F |
| --- | --- | --- | --- | --- | --- |
| Model | 5 | 1,0359E-6 | 2,07181E-7 | 153,19002 | 1,93612E-10 |
| Error | 12 | 1,62293E-8 | 1,35244E-9 |  |  |
| Total | 17 | 1,05213E-6 |  |  |  |
